# Neuropeptide S Attenuates the Alarm Pheromone-Evoked Defensive and Risk Assessment Behaviors Through Activation of Cognate Receptor-Expressing Neurons in the Posterior Medial Amygdala

**DOI:** 10.3389/fnmol.2021.752516

**Published:** 2021-12-24

**Authors:** Yu-Feng Shao, Can Wang, Xiao-Ping Rao, Hua-Dong Wang, Yan-Li Ren, Jing Li, Chao-Yu Dong, Jun-Fan Xie, Xing-Wen Yang, Fu-Qiang Xu, Yi-Ping Hou

**Affiliations:** ^1^Departments of Neuroscience, Anatomy, Histology, and Embryology, Key Laboratory of Preclinical Study for New Drugs of Gansu Province, School of Basic Medical Sciences, Lanzhou University, Lanzhou, China; ^2^Key Lab of Neurology of Gansu Province, Lanzhou University, Lanzhou, China; ^3^Center of Brain Science, State Key Laboratory of Magnetic Resonance and Atomic and Molecular Physics, National Center for Magnetic Resonance in Wuhan, Key Laboratory of Magnetic Resonance in Biological Systems, Innovation Academy for Precision Measurement Science and Technology, Chinese Academy of Sciences, Wuhan, China; ^4^Shenzhen Key Lab of Neuropsychiatric Modulation and Collaborative Innovation Center for Brain Science, Guangdong Provincial Key Laboratory of Brain Connectome and Behavior, CAS Key Laboratory of Brain Connectome and Manipulation, Brain Cognition and Brain Disease Institute (BCBDI), Shenzhen Institute of Advanced Technology, Chinese Academy of Sciences, Shenzhen-Hong Kong Institute of Brain Science-Shenzhen Fundamental Research Institutions, Shenzhen, China; ^5^Wuhan National Laboratory for Optoelectronics, Huazhong University of Science and Technology, Wuhan, China; ^6^Center for Excellence in Brain Science and Intelligence Technology, Chinese Academy of Sciences, Shanghai, China

**Keywords:** neuropeptide S, neuropeptide S receptor, alarm pheromone, neural circuit tracing, herpes simplex virus, posterior medial amygdala, antagonist, c-Fos

## Abstract

Neuropeptide S (NPS) acts by activating its cognate receptor (NPSR). High level expression of NPSR in the posterior medial amygdala suggests that NPS-NPSR system should be involved in regulation of social behaviors induced by social pheromones. The present study was undertaken to investigate the effects of central administration of NPS or with NPSR antagonist on the alarm pheromone (AP)-evoked defensive and risk assessment behaviors in mice. Furthermore, H129-H8, a novel high-brightness anterograde multiple trans-synaptic virus, c-Fos and NPSR immunostaining were employed to reveal the involved neurocircuits and targets of NPS action. The mice exposed to AP displayed an enhancement in defensive and risk assessment behaviors. NPS (0.1–1 nmol) intracerebroventricular (i.c.v.) injection significantly attenuated the AP-evoked defensive and risk assessment behaviors. NPSR antagonist [D-Val^5^]NPS at the dose of 40 nmol completely blocked the effect of 0.5 nmol of NPS which showed the best effective among dose range. The H129-H8-labeled neurons were observed in the bilateral posterodorsal medial amygdala (MePD) and posteroventral medial amygdala (MePV) 72 h after the virus injection into the unilateral olfactory bulb (OB), suggesting that the MePD and MePV receive olfactory information inputs from the OB. The percentage of H129-H8-labeled neurons that also express NPSR were 90.27 ± 3.56% and 91.67 ± 2.46% in the MePD and MePV, respectively. NPS (0.5 nmol, i.c.v.) remarkably increased the number of Fos immunoreactive (-ir) neurons in the MePD and MePV, and the majority of NPS-induced Fos-ir neurons also expressed NPSR. The behavior characteristic of NPS or with [D-Val^5^]NPS can be better replicated in MePD/MePV local injection within lower dose. The present findings demonstrated that NPS, via selective activation of the neurons bearing NPSR in the posterior medial amygdala, attenuates the AP-evoked defensive and risk assessment behaviors in mice.

## Introduction

In their continuous struggle for survival, predator and prey species interact to maximize the likelihood of finding food or avoiding being eaten. Preys have adjusted to deal with dangerous encounters using specialized sensory systems (Brechbühl et al., 2013). Alarm pheromone (AP), released from conspecific animal who is under the threatening situation (Ono et al., 2003; Brechbühl et al., 2008, 2013), relays the presence of danger and possibly plays an important role in increasing overall species fitness (Kiyokawa et al., 2006; Brechbühl et al., 2008). AP usually induces conspecifics to increase freezing, escaping, restless and body temperature (Kikusui et al., 2001; Kiyokawa et al., 2005; Brechbühl et al., 2008; Inagaki et al., 2014) and has been referred to as a stress-related odor (Kikusui et al., 2001; Kiyokawa et al., 2006; Inagaki et al., 2014) based on it-induced behavioral responses. In terms of chemical properties, AP is volatile, hydrophilic, and short-lived molecules, and its main active components include 2-sec-butyl-4,5-dihydrothiazole (SBT), 4-methylpentanal and hexanal which have been, respectively, demonstrated as the anxiogenic molecules to enhance anxiety-like behaviors (Brechbühl et al., 2013; Inagaki et al., 2014).

In rodents, the vomeronasal organ (VNO) (Kiyokawa et al., 2013) and Grüeneberg ganglion (GG) (Brechbühl et al., 2008) of olfactory organs have been identified to detect AP. The VNO (also called “Jacobson’s organ”) belongs to accessory olfactory system (AOS), and is a tubular structure that lies at the base of the nasal septum (Wysocki, 1979). The vomeronasal sensory neurons (VSNs) are distributed in the vomeronasal epithelium of the VNO, and their targets are in glomeruli in the accessory olfactory bulb (AOB) (Firestein, 2001; Halpern and Martinez-Marcos, 2003; Yokosuka, 2012). The secondary and tertiary centers of the VNO include the bed nucleus of stria terminalis (BNST), bed nucleus of the accessory olfactory tract, the posteroventral medial amygdala (MePV) and posterodorsal medial amygdala (MePD) (Mohedano-Moriano et al., 2007; Yokosuka, 2012; Zheng et al., 2020). In contrast, the GG is a part of main olfactory system (MOS), and is an arrow-shaped neuronal structure and situates underneath the dorsal cartilage of the anterior tip of the nasal cavity. Their axons’ targets locate outside but around the rostral part of the AOB (Fuss et al., 2005; Koos and Fraser, 2005; Roppolo et al., 2006). Recent studies have demonstrated that the GG neurons were activated, respectively, by SBT (Brechbühl et al., 2013; Chao et al., 2018) and by 2-propylthietane (2-PT) released from the stoat anal gland (Pérez-Gómez et al., 2015). Although the higher center of the GG is still unclear, exposure of mice to 2-PT increases the number of Fos-immunoreactive neurons in the MePV (Pérez-Gómez et al., 2015). Thus, that the olfactory signals from the MOS and AOS converge on the MePD and MePV of medial amygdaloid nucleus (MeA) should be involved in regulation of social behaviors induced by social pheromones (Keshavarzi et al., 2015; Matsuo et al., 2015).

Neuropeptide S (NPS) and its cognate receptor (NPSR) system is a newly identified neuromodulator system (Xu et al., 2004, 2007; Reinscheid et al., 2005; Clark et al., 2011). NPS precursor messenger ribonucleic acid (mRNA) in mouse is expressed in the Kölliker-Fuse nucleus and pericoerulear area of the brainstem (Clark et al., 2011). In contrast to NPS precursor mRNA, NPSR or its precursor mRNA is widely distributed in mouse brain, mainly in the olfactory cortex, cerebral cortex, thalamus, hypothalamus, subiculum complex of hippocampal formation and amygdala including basolateral amygdaloid nucleus (BLA), cortical amygdaloid nucleus (CoA), and MeA (Xu et al., 2004, 2007; Clark et al., 2011; Leonard and Ring, 2011; Shao et al., 2013, 2016; Wang et al., 2020).

This profile of NPSR expression suggests the involvement of NPS-NPSR system in the regulation of multiple central functions. For example, specific activation of NPSR by central administration of NPS promotes wakefulness in rats (Xu et al., 2004; Zhao et al., 2012; Kong et al., 2017), and enhances locomotor and exploratory activities, and evokes anxiolytic-like effects in mice (Xu et al., 2004; Duangdao et al., 2009; Enquist et al., 2012; Xie et al., 2018), and inhibits food intake in mice and rats (Beck et al., 2005; Smith et al., 2006; Fedeli et al., 2009; Cifani et al., 2011; Shao et al., 2013), and ameliorates cognition in mice (Zhao et al., 2019), and facilities olfactory function (Shao et al., 2013) and olfactory spatial memory in mice (Shao et al., 2016; Wang et al., 2020). The high NPSR expression in the MeA suggests that NPS-NPSR system is involved in the regulation of AP-induced defensive and risk assessment behaviors.

Therefore, the present study was designed to examine the effect of NPS-NPSR system on AP-induced the defensive and risk assessment behaviors using the modified open-field apparatus described by Kiyokawa et al. (2005). The anterograde multisynaptic neural circuits tracing from the olfactory bulb (OB) to the posterior medial amygdala were mapped with the novel high-brightness viral tracer H129-H8 (Su et al., 2020). To further identify the neuronal targets of NPS in the MePD and MePV, NPS-induced Fos-immunereactive (-ir) neurons were analyzed using *ex vivo* immunohistochemistry, and the co-presence of NPSR in H129-H8-labeled and Fos-ir neurons were examined using dual-immunofluorescence microscopy.

## Materials and Methods

### Animals and Surgical Implantation

Adult male C57BL/6J mice (6 weeks ≤ age < 12 weeks old, *n* = 91) were purchased from Experimental Animal Central of Lanzhou University, not including the mice for anterograde trans-synaptic circuit tracing. They were housed in plastic cages (290 mm L × 165 mm W × 130 mm H, 4–5 mice per cage) with ambient temperature (22 ± 1°C) and a relative humidity of 50% on an automatically controlled 12:12-h light/dark cycle (lights on 8:00–20:00 h, illumination intensity ≈ 100 lux). Food and water were available *ad libitum*. All animals were cared for and experiments were conducted in accordance with the National Institutes of Health Guide for the Care and Use of Laboratory Animals (2011 revision). The experimental protocol was approved by the Ethics Committee of Lanzhou University (permit number: SCXK Gan 2013-0002 and 2018-0002).

Under chloral hydrate anesthesia (350 mg/kg, i.p.), mice were placed in a stereotaxic apparatus (Item: 68030, RWD, Shenzhen, China). A stainless-steel guide cannula (25 gauge) was stereotaxically implanted above the right lateral ventricle (AP –0.2 mm, ML + 1.0 mm, DV –1.4 mm) or bilateral posterior medial amygdala (AP –1.55 mm, ML ± 1.90 mm, DV –4.25 mm, according to the atlases of Paxinos and Franklin, 2001) for intracerebroventricular (i.c.v.) and local injection, respectively. Cannula was chronically fixed to skull with dental cement. A stainless-steel indwelling stylet (32 gauge) was inserted into the guide cannula to prevent occlusion.

### Alarm Pheromone Collection

**FIGURE 1:**
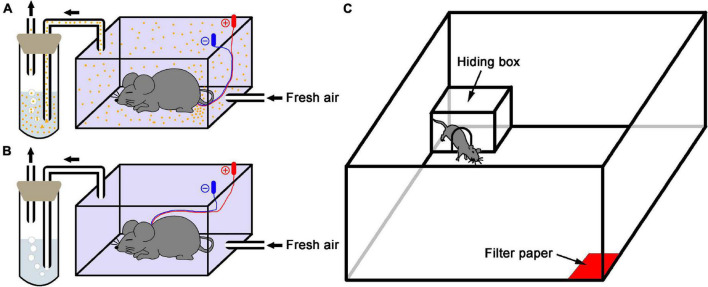
Schematic diagram of the experimental setup. **(A)** Alarm pheromone collection. An anesthetized donor mouse in the sealed plastic box received electrical stimulation of the perianal region, and the fresh air carried alarm pheromone (orange dots) into the purified water. **(B)** Control collection. A mouse received electrical stimulation of the subcutaneous part of the napex as vehicle because the donor mouse didn’t release AP in this condition. **(C)** The test apparatus was used in this study.

The collection method of AP used in the present study was modified as previously described by Kiyokawa et al. (2005). Briefly, a donor mouse under chloral hydrate anesthesia (350 mg/kg, i.p.) was implanted with two intradermal needles (25 gauge) at the edge of both sides of the anal canal for electrical stimulation of the perianal region and then placed into a sealed plastic box (15 cm L × 7.5 cm W × 10 cm H) for 5 min. During this period, the donor mouse received electrical stimuli of 10 V (1.5 mA approximately) for 1 s at 20 s intervals. Meanwhile, compressed fresh air was constantly (100 mL/min) flowed into the box through an inlet port at its bottom and pushed the gaseous contents containing the AP released from the donor mouse into 5 mL purified water within a cuvette via a tygon tubing installed at the top of the box. Finally, the hydrophilic and volatile AP trapped in water were collected (Figure 1A). For vehicle collection, the procedure was similar to the above except the stimulating electrodes placed at the subcutaneous part of the napex in subject mice (Figure 1B). After collection, the AP-containing and vehicle water were stored at 4^°^C for 3 h until use.

### Drug Administrations

NPS (mouse, Ser-Phe-Arg-Asn-Gly-Val-Gly-Ser-Gly-Ala-Lys-Lys-Thr-Ser-Phe-Arg-Arg-Ala-Lys-Gln) and [D-Val^5^]NPS (human, Ser-Phe-Arg-Asn-D-Val-Val-Gly-Thr-Gly-Met-Lys-Lys-Thr-Ser-Phe-Gln-Arg-Ala-Lys-Ser) were purchased from Shanghai Mocell Biotech Co., Ltd. Fresh NPS or NPS + [D-Val^5^]NPS was dissolved in saline (1 μL for i.c.v. injection and 0.3 μL for local injection) and, respectively, administered 5 min before behavior test through the planted guide cannula with the flow rate 1 or 0.3 μL/min. Corresponding with drugs injection, i.c.v. or local injection saline were used as the control in the present study.

### Behavioral Test

Behavioral experiments were performed in a modified open field (100 cm L × 100 cm W × 40 cm H) as previously described by Kiyokawa et al. (2006). Briefly, a filter paper (5 × 5 cm) soaked with 650 μL of either AP-containing or vehicle water was placed in one corner. Each subject mouse was exposed to new filter papers soaked with either type of water that was prepared by the independent preparation procedure. The experimental field was illuminated at about 100 lux. All subject mice were individually placed in the center of arena and allowed to explore the arena and perceive the stimuli for 5 min for acclimation. In behavior test, a polycarbonate hiding box (17.5 cm L × 24.5 cm W × 12.5 cm H) with a gate (7.5 cm diameter) in one wall was set in the corner opposite to the stimuli. A subject mouse received a drug or saline 5 min prior to test was placed in center of arena and exposed to a stimulus of either AP or vehicle, and the behavioral parameters including the moving distance and time in open arena, the time in hiding box and time of head-out through gate were recorded by a video camera connected with a computer-operated motion tracking system (R. D. BehaviorSys v2.8.7, Mobiledatum, Shanghai, China) for 10 min (Figure 1C). In addition, subject mice had been accustomed to the box in their home cage for 24 h since the day before the experiment. The box was cleaned with an alcohol paper towel before and between experiments if a subject urinated or defecated in box. After the experiments, the open field was cleaned with ethanol and paper towels, and the hiding box was thoroughly washed in hot water with a cleanser for subsequent uses. Behavior experiments were carried out between 09:00 and 12:00 h to reduce the influences of circadian rhythm.

When an experiment was over, mice were i.c.v. injected with 1 μL methylene blue dye through guide cannula and were decapitated under deep anesthesia with over dose of chloral hydrate (400 mg/kg) 5 min later. Brains were removed and frozen at –20^°^C. Gross dissection of the brain was carried out to verify the site of drugs or vehicle administration. Only data from animals with dye dispersion throughout the ventricle were used (Shao et al., 2013, 2016).

### Anterograde Trans-Synaptic Circuit Tracing

All procedures were performed in Biosafety Level-2 (BSL-2) laboratory and animal facilities, and were conducted in accordance with the guidelines of the Animal Care and Use Ethics Committees at the Innovation Academy for Precision Measurement Science and Technology, Chinese Academy of Sciences.

#### Surgery and Viral Injection

Adult C57BL/6J mice (*n* = 10), purchased from Hunan SJA Laboratory Animal Company, were anesthetized with pentobarbital sodium (80 mg/kg, i.p.), and placed in a stereotaxic apparatus (Item: 68030, RWD, Shenzhen, China). During surgery and virus injection, all animals were kept anesthetized with isoflurane (1–1.5%). The skull above the targeted area was thinned with a dental drill and removed carefully. Injections were conducted with a syringe pump (Item: 53311, Quintessential stereotaxic injector, Stoelting, Wood Dale, IL, United States) connected to a glass micropipette (φ = 10–15 μm) with a speed at 10 nL/min. The glass micropipette was held for an extra 10 min after the completion of the injection and then slowly retreated. After the surgery, the incisions were stitched, lincomycin hydrochloride and lidocaine hydrochloride gel were applied to prevent inflammation and alleviate pain for the animals.

To trace the multiple synaptic efferent of the OB, a novel high-brightness tracer H129-H8 (rH129-CMV-Rep/ITR-hUbC-EGFP-WPRE-ITR, 100 nL, 6 × 10^5^ plaque-forming units/mL) (Su et al., 2020) was unilaterally injected into the OB (AP + 4.28 mm, ML + 0.50 mm, DV –2.20 mm).

#### Tissue Preparation and Dual-Immunofluorescences With NPSR

Seventy-two hours after H129-H8 injection, mice were anesthetized with pentobarbital sodium (100 mg/kg, i.p.), and perfused transcardially with 30 mL sodium phosphate buffer (PBS) and followed by ice-cold 4% paraformaldehyde (PFA) dissolved in 0.1 M PBS. The brain tissues were carefully removed and post-fixed in PBS containing 4% PFA at 4°C overnight, and then equilibrated in PBS containing 30% sucrose at 4°C for 3 days. The 40 μm thick coronal sections were obtained using the cryostat microtome (Thermo, CRYOSTAR NX50, Cheshire, United Kingdom). Sections containing MePD/MePV were incubated with goat polyclonal antibody against NPSR (1:1,000, sc-162893, Santa Cruz Biotechnology, Santa Cruz, CA, United States) diluted in PBS containing 1% bovine serum for 48 h at 4°C on an agitator after incubation in 10% bovine serum in PBS. After several rinses in PBS, sections were incubated with CyTM 3-conjugated affinipure donkey anti-goat IgG (1:1,000, 705-165-147, Jackson ImmunoResearch Laboratories, Inc., PA, United States) for 4 h at room temperature and then with DAPI (1:2000, D8417, Sigma-Aldrch, Saint Louis, MO, United States) for 10 min. Finally, sections were on slides, covered with a coverslip, mounted with 70% glycerol (in PBS) and sealed with nail polish. Images were captured with the virtual microscopy slide-scanning system (VS120, Olympus, Tokyo, Japan).

### Immunohistochemistry

#### Tissue Preparation

One hour and a half after NPS (0.5 nmol, *n* = 5) or saline 1 μL (*n* = 6) i.c.v. administration, animals were anesthetized with over dose chloral hydrate (400 mg/kg), and perfused via the ascending aorta with 30 mL saline containing heparin (1 U/mL) and followed by 4% PFA in 0.1 M phosphate buffer (PB). Brains were removed, post-fixed in the same fixative overnight and immersed in 30% sucrose solution in 0.1 M PB at 4°C until the samples dewatering completely. The 40 μm thick coronal slices were obtained using the cryostat microtome (CM1900, Leica Micro-systems, Heidelberg, Germany) and stored at –20°C.

#### Fos Immunohistochemistry

The floating sections were rinsed in 0.01 M PBS (pH 7.4), treated 30 min in 0.3% H_2_O_2_ in PBS, and incubated in blocking solution (10% bovine serum in PBS) for 1 h. Sections were incubated with a rabbit polyclonal antibody against c-Fos (1:5,000, sc-253, Santa Cruz Biotechnology, Santa Cruz, CA, United States) diluted in PBS containing 1% bovine serum for 48 h at 4°C on an agitator. After rinsing in PBS, sections were incubated with a biotinylated goat anti-rabbit IgG (1:1,000, AP132B, Millipore, Temecula, CA, United States) then with horseradish peroxidase conjugated streptavidin (1:2,000, S2438, Sigma-Aldrich, Saint Louis, MO, United States). Both incubations were placed on an agitator at 4°C overnight. Following rinsing, the sections were immersed in 0.05 M Tris-HCl buffer, pH 7.6, containing 0.05% 3,3’diaminobenzidine (DAB), 0.01% H_2_O_2_, and 0.6% nickel ammonium sulfate for 2–5 min at room temperature. Finally, the sections were mounted on gelatin-coated glass slides, processed with counter-staining with neutral red, dried, dehydrated, and covered with a cover slip, using DPX, for light microscopy.

#### Dual-Immunofluorescences for Fos and NPSR

These sections were incubated with mixture solution including rabbit polyclonal antibody against c-Fos (1:1,000, sc-253, Santa Cruz Biotechnology, Santa Cruz, CA, United States) and goat polyclonal antibody against NPSR (1:1,000, sc-162893, Santa Cruz Biotechnology, Santa Cruz, CA, United States) diluted in PBS containing 1% bovine serum for 48 h at 4°C on an agitator after incubation in 10% bovine serum in PBS. The specificity of the anti-NPSR antibody has been demonstrated in previous studies (Laitinen et al., 2004; Vendelin et al., 2005; Shao et al., 2013, 2016; Xie et al., 2018; Wang et al., 2020). After several rinses in PBS, sections were incubated with Alexa Fluor^^®^^ 488-conjugated affinipure donkey anti-rabbit IgG (1:1,000, 711-545-152, Jackson ImmunoResearch Laboratories, Inc., PA, United States) and CyTM 3-conjugated affinipure donkey anti-goat IgG (1:1,000, 705-165-147, Jackson ImmunoResearch Laboratories, Inc., PA, United States) for 4 h at room temperature. Finally, sections were mounted on slides, covered with a coverslip, using 90% glycerol in 0.1 M PB, and observed under a fluorescence microscope (BX53, Olympus, Tokyo, Japan) and photographed under Zeiss LSM 710 laser confocal microscope.

### Data Analysis

#### Behavioral Assay

The behavioral parameters including “walking distance” (the total distance traveled outside the hiding box during the recording time), “outside” (the time of the mouse spent in the open arena), “conceal” (the time of the mouse being entirely inside the hiding box) and “head-out” (the time of the mouse poking its head, or head and shoulders, out of the hiding box entrance with their hind paws remaining inside the box) were collected and analyzed by a researcher who was blind to the experiment. Heatmap plots in Supplementary Figures 1, 2 were calculated by addition of the trajectories of all mice in each group.

#### Cell Counting

Sections containing MePD and MePV (Bregma –1.34, –1.58, –1.82, and –2.06 mm) were observed and photographed under light microscopy. The areas of the MePD and MePV were determined by the characteristics of their cytoarchitecture and peripheral white matter according to the atlas of Paxinos and Franklin (2001). Fos-ir neurons were bilaterally counted with counting tool of ZEISS Efficient Navigation (ZEN) microscope software (Germany) of each animal treated with NPS or saline. The total value for two sides was calculated.

#### Statistical Analysis

All data were expressed as means ± SEM. Different parameters were analyzed using independent Student’s *t*-test or one-way analysis of variance (ANOVA) and the Tukey-Kramer’s multiple comparisons test. In all statistical comparisons, the level of significances was set at *P* < 0.05.

## Results

### Alarm Pheromone Evoked Defensive and Risk Assessment Behaviors

**FIGURE 2:**
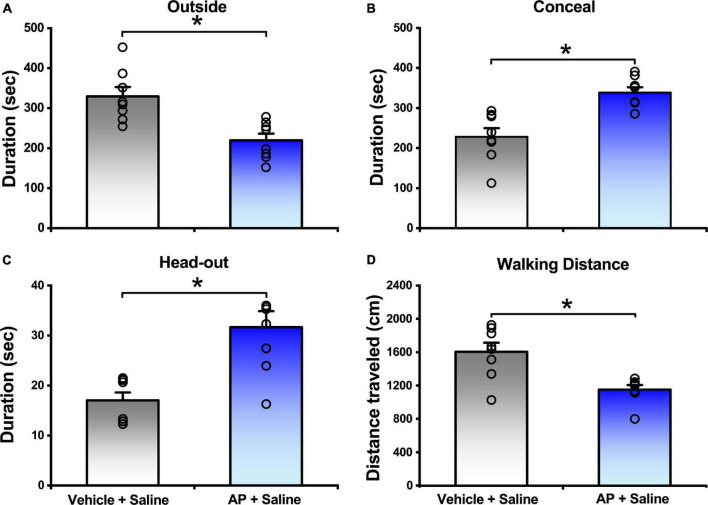
Behavior responses of mice that were either exposed to AP or vehicle. Saline were i.c.v. injection. Values are means ± SEM (*n* = 8 in each group). *P < 0.01. Data were analyzed by independent Student’s *t*-test.

In comparison with the mice responded to vehicle, the mice exposed to AP significantly reduced the outside duration (219.70 ± 16.51 s vs. 329.63 ± 23.04 s, *P* < 0.01, Figure 2A) and walking distance (1152.80 ± 53.75 cm vs. 1605.31 ± 107.97 cm, *P* < 0.01, Figure 2D and Supplementary Figure 1), whereas they significantly increased the concealment duration (338.50 ± 13.12 s vs. 228.26 ± 21.38 s, *P* < 0.01, Figure 2B) and head-out duration (31.68 ± 3.19 s vs. 17.02 ± 1.58 s, *P* < 0.01, Figure 2C).

### Neuropeptide S Attenuated the Alarm Pheromone-Evoked Defensive and Risk Assessment Behaviors

**FIGURE 3:**
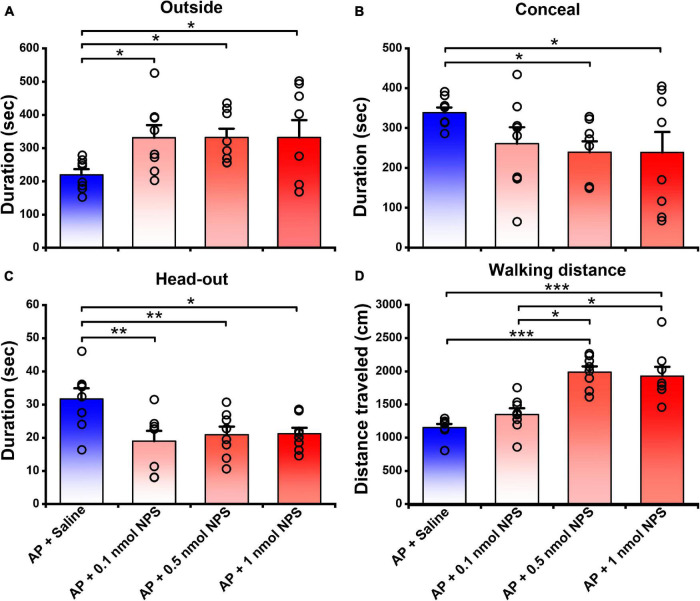
Attenuating effects of NPS on the AP-induced defensive and risk assessment behaviors in mice. Saline or NPS were i.c.v. injection. AP + Saline group is same as in Figure 2. Values are means ± SEM (*n* = 8 in each group). *P < 0.05, **P < 0.01, ***P < 0.001. Data were analyzed by one-way ANOVA and Tukey-Kramer’s multiple comparisons test.

In the presence of AP condition, the mice i.c.v. given 0.1, 0.5, and 1 nmol of NPS relative to the mice given saline increased the outside duration (Figure 3A) from 219.70 ± 16.51 s, respectively, to 331.36 ± 37.50 s (*P* < 0.05), 331.62 ± 27.09 s (*P* < 0.05) and 332.02 ± 52.25 s (*P* < 0.05) and walking distance (Figure 3D and Supplementary Figure 1) from 1152.80 ± 53.75 cm, respectively, to 1348.47 ± 93.42 cm (*P* = 0.166), 1989.82 ± 85.19 cm (*P* < 0.001) and 1927.60 ± 137.64 cm (*P* < 0.001), and reduced the concealment duration (Figure 3B) from 338.50 ± 13.12 s, respectively, to 260.92 ± 41.40 (*P* = 0.143), 239.45 ± 27.36 s (*P* < 0.05) and 238.66 ± 51.72 (*P* < 0.05), and head-out duration (Figure 3C) from 31.68 ± 3.19 s, respectively, to 18.75 ± 3.07 s (*P* < 0.01), 20.90 ± 2.40 s (*P* < 0.01) and 21.16 ± 1.79 s (*P* < 0.05). Statistical analyses revealed that the walking distance increased by 0.5 and 1 nmol of NPS was greater than that by 0.1 nmol of NPS (*P* < 0.001, Figure 3D and Supplementary Figure 1). These results indicated that NPS enables to allay the AP-evoked anxiety-like behaviors, and that the best effective dose is 0.5 nmol which significantly and completely attenuates the AP-induced defensive and risk assessment behaviors.

### [D-Val^5^]NPS Antagonized the Attenuating Effects of Neuropeptide S on the Alarm Pheromone-Evoked Defensive and Risk Assessment Behaviors

**FIGURE 4:**
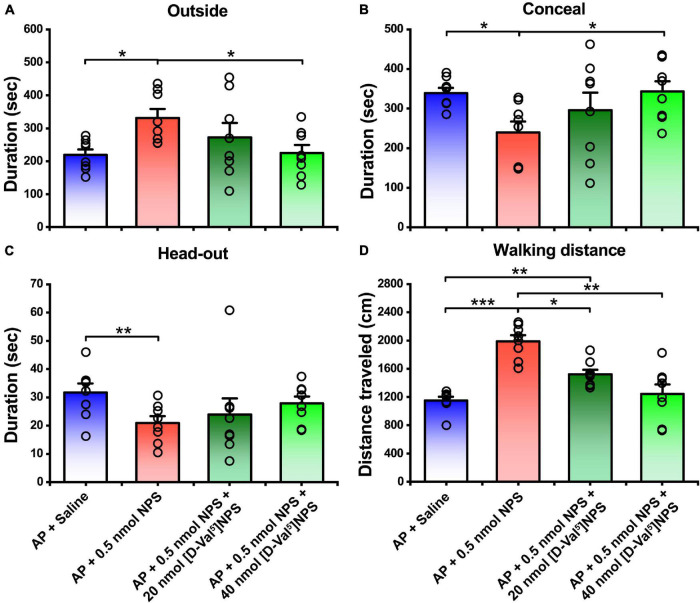
Attenuating effects of NPS on the AP-induced defensive and risk assessment behaviors were blocked by [D-Val^5^]NPS. Saline, NPS or NPS + [D-Val^5^]NPS were i.c.v. injection. AP + Saline and AP + 0.5 nmol NPS groups are same as in Figures 2, 3. Values are means ± SEM (*n* = 8 in each group). *P < 0.05, **P < 0.01, ***P < 0.001. Data were analyzed by one-way ANOVA and Tukey-Kramer’s multiple comparisons test.

To clear whether the attenuating effects of NPS on the AP-evoked defensive and risk assessment behaviors are blocked by NPSR antagonist, [D-Val^5^]NPS, a selective antagonist of NPSR (Guerrini et al., 2009; Shao et al., 2013, 2016; Xie et al., 2018; Wang et al., 2020), was i.c.v. administrated with NPS to the mice subjected to AP. The results showed that both 20 and 40 nmol of [D-Val^5^]NPS attenuated the effects of 0.5 nmol NPS on the AP-evoked defensive and risk assessment behaviors, and high dose of [D-Val^5^]NPS displayed more effective than low dose (Figure 4 and Supplementary Figure 1).

### NPSR Positive Neurons in the Medial Amygdala and Medial Amygdala Received Informational Inputs From Olfactory Bulb

**FIGURE 5:**
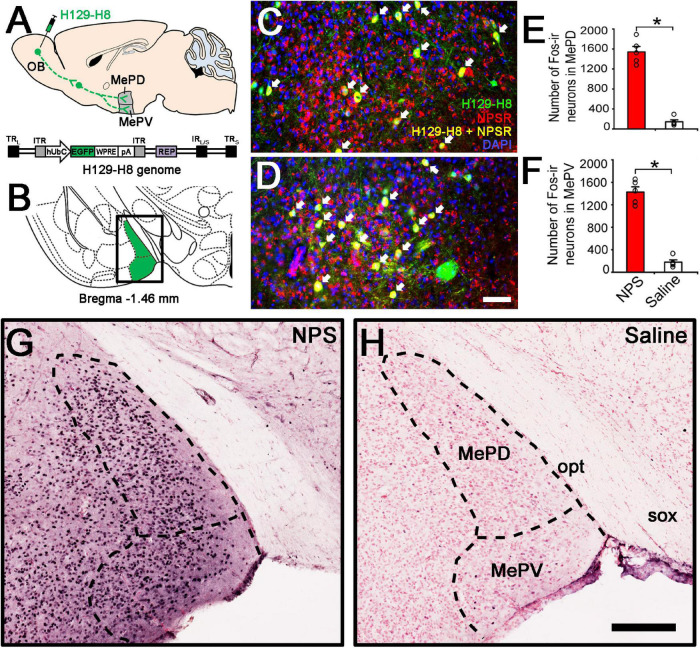
Anterograde viral tracing and NPS-induced the activated neurons in posterior medial amygdala. **(A)** Schematic diagram of H129-H8 injection. **(B)** Schematic drawing shows the section illustrated in **(C,D,G,H)**. **(C,D)** Ipsilateral coronal section of a H129-H8 (green) treated mouse brain at the MePD **(C)** and MePV **(D)** stained with NPSR (red) and DAPI (blue). **(E,F)** Histograms show quantitative analysis of the number of Fos-ir neurons in the MePD and MePV following NPS (*n* = 5) and saline (*n* = 6) i.c.v. injection, respectively. **(G,H)** Photomicrographs show Fos-ir neurons (black) in the MePD and MePV in NPS- and saline-treated mice, respectively. Values are means ± SEM. *P < 0.001. Data were analyzed by independent Student’s *t*-test. Arrow **(C,D)** show the co-expression of H129-H8-labled and NPSR-ir neurons. Bar = 100 μm **(C,D)**, 200 μm **(G,H)**. OB, olfactory bulb; MePD, posterodorsal medial amygdaloid nuclei; MePV, posteroventral medial amygdaloid nuclei; opt, optic tract; sox, supraoptic decussation.

To investigate whether there is an anterograde multisynaptic neural circuit from the OB to the posterior medial amygdala where is well-kwon to play a key role in the social behaviors induced by pheromones (Matsuo et al., 2015; Pérez-Gómez et al., 2015). When H129-H8 virus, an anterograde high-brightness tracer, was used to map the neural circuits, its positive neurons were found in the bilateral MePD and MePV of MeA (Figures 5A–D) 72 h after the virus injection into the unilateral OB of mice. The percentage of H129-H8-labeled neurons that also express NPSR were 90.27 ± 3.56% and 91.67 ± 2.46% in the MePD and MePV, respectively (Figures 5C,D).

### Neuropeptide S Activated Fos Expression in the Medial Amygdala and Medial Amygdala

To identify whether the neurons in the MePD and MePV are activated by NPS, c-Fos immunohistochemistry was employed to label the NPS-induced activation neurons. Following i.c.v. administration of NPS (0.5 nmol), dense Fos-ir neurons were seen in the posterior medial amygdala including the MePD and MePV (Figure 5G), whereas a few Fos-ir neurons were found in the same areas after saline administration (Figure 5H). The statistical analyses showed that NPS significantly increased the number of Fos-ir neurons in the MePD (1542.40 ± 108.38 vs. 143.60 ± 38.28, *P* < 0.001, Figure 5E) and MePV (1426.80 ± 92.72 vs. 179.2 ± 40.50, *P* < 0.001, Figure 5F) compared with saline.

### Neuropeptide S-Induced Fos-Immunereactive Neurons in the Medial Amygdala and Medial Amygdala Expressed NPSR

**FIGURE 6:**
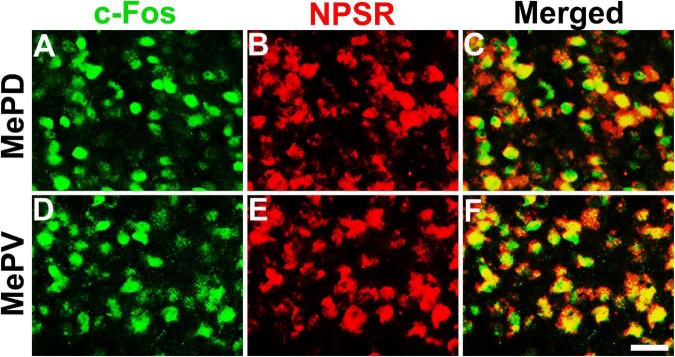
NPS-induced Fos-ir neurons bearing NPSR in the MePD and MePV of posterior medial amygdala. Photomicrographs show Fos-ir neurons in the MePD **(A)** and MePV **(D)** after NPS i.c.v. administration, NPSR-ir neurons in the MePD **(B)** and MePV **(E)**, and the co-expression of Fos-ir and NPSR-ir neurons in the MePD **(C)** and MePV **(F)**, respectively. Yellow **(C,F)** show the co-expression of Fos-ir and NPSR-ir neurons. Bar = 50 μm.

To determine whether the NPS-induced Fos-ir neurons in the MePD and MePV express NPSR or not, Fos-ir staining combined with NPSR-ir staining assay was performed. As shown in Figure 6, the percentage of Fos-ir neurons that also perform NPSR staining positive was 81.84 ± 2.58% in the MePD (Figures 6A–C) and 87.46 ± 0.86% in the MePV (Figures 6D–F), respectively.

### Local Injection of Neuropeptide S or With [D-Val^5^]NPS in the Medial Amygdala/Medial Amygdala Replicated the Behavior Characteristic of i.c.v. Injection

**FIGURE 7:**
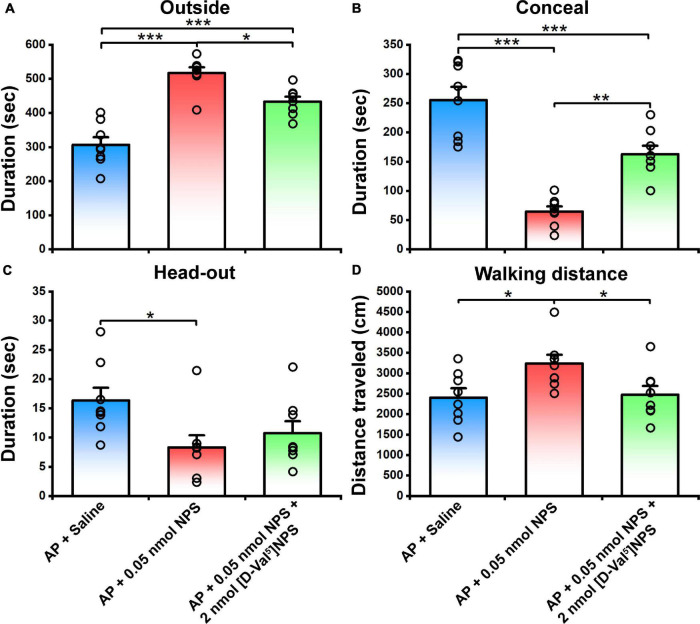
Defensive and risk assessment behavioral test following MePD/MePV local injection of Saline, NPS or NPS + [D-Val^5^]NPS. Values are means ± SEM (*n* = 8 in each group). *P < 0.05, **P < 0.01, ***P < 0.001. Data were analyzed by one-way ANOVA and Tukey-Kramer’s multiple comparisons test.

To verify whether the behavior characteristic of NPS-NPSR system are exerted through MePD and MePV, NPS (0.05 nmol) or NPS (0.05 nmol) + [D-Val^5^]NPS (2 nmol) was local administrated to the mice subjected to AP. The results showed that 0.05 nmol of NPS attenuated the AP-evoked defensive and risk assessment behaviors, and these behaviors were blocked by co-injection of [D-Val^5^]NPS (2 nmol) (Figure 7 and Supplementary Figure 2).

## Discussion

The present study demonstrated that central action of NPS reversed the increased defensive (concealment) and risk assessment (head-out) behaviors, and the reduced exploratory (outside duration and walking distance in open arena) behaviors evoked by AP (Figures 2, 3, and Supplementary Figure 1). Noticeably, 0.1 nmol of NPS significantly attenuated the increased head-out (Figures 2C, 3C) and decreased outside durations (Figures 2A, 3A) of the mice exposed to AP, but it did not significantly affect the other behaviors although it tended to reduce concealment (Figure 3B), suggesting that the minute amount of NPS enables the stressed animals to alleviate the risk assessment behavior and partially improve defensive and exploratory behaviors, but it is not yet enough to enable them to move freely in open arena like control animals (Figures 2D, 3D, and Supplementary Figure 1). Actually, the dosage of 0.5 nmol of NPS is enough to attenuate the AP-evoked anxiety-related behaviors. AP is a stress-related odor containing anxiogenic molecules and elicits the innate and stereotypic anxiety-related behaviors in conspecifics, such as defensive (e.g., escape into a burrow in wild or small hiding box in laboratory) and risk assessment (e.g., typical “head-out” and flat back) behaviors (Kiyokawa et al., 2006; Brechbühl et al., 2013; Inagaki et al., 2014). NPS, a recently discovered neuromodulator, presents a beneficial therapeutic candidate due to its potent anxiolytic activity (Xu et al., 2004; Pape et al., 2010; Grund et al., 2017). For instance, NPS and NPSR knockout mice exhibit anxiety-like behavior (Duangdao et al., 2009; Liu et al., 2017). Central administration of NPS counteracts the anxiety-like behaviors induced, respectively, by persistent nociception (Zhang et al., 2014) and by paradoxical sleep deprivation (Xie et al., 2018). Sustained overexpression of NPS in the amygdala reduces anxiety-like behavior (Donner et al., 2010). NPS-NPSR signaling is also involved in human anxiety (Donner et al., 2010). Thus, our data provide the first evidence that NPS attenuates the anxiety-related behaviors evoked by offensive odorants, and accordingly, our findings raise the intriguing possibility that NPS is a beneficial therapeutic candidate for anxiolysis.

More importantly, our study also aims at the anatomical pathway involved in the AP-induced anxiety-related behaviors and the potential targets of NPS. The OB is the gate and the first relay station of olfactory system and summates olfactory signals including AP from olfactory receptors and then effectively transfers them to olfactory cortex and several subcortical regions to regulate behavioral states and cognitive functions (Peretto et al., 2014; Carew et al., 2018). The MePD and MePV of MeA are key nodes in anxiety-related and social behavioral responses induced by pheromones (Keshavarzi et al., 2015; Matsuo et al., 2015). In our study, when H129-H8, a novel high-brightness anterograde multiple trans-synaptic virus modified from herpes simplex virus type 1 (HSV-1) strain H129 (Su et al., 2020), was injected into the unilateral OB, its label neurons were found in the bilateral MePD and MePV (Figure 5C). The result indicates that the MePD and MePV receive the inputs from the bilateral OB. The percentage of H129-H8-labeled neurons that also express NPSR were 90.27 ± 3.56% and 91.67 ± 2.46% in the MePD and MePV, respectively (Figures 5C,D). Furthermore, the identification of the potential targets of NPS were examined by neurons expressing Fos, the product of the immediate early gene that is expressed in association with neuronal activation (Morgan and Curran, 1986; Dragunow and Faull, 1989), we found that NPS treatment markedly increased the number of Fos-ir neurons in the MePD and MePV compared with saline (Figures 5G,H). The evidence points that NPS enhances the activation of neurons in the MePD and MePV to presumably exert the anxiolytic effect on the AP-evoked anxiety-related behaviors. Indeed, the growing experimental evidences have revealed that the MePD and MePV of MeA are involved in social and defensive behaviors (Dielenberg et al., 2001; McGregor et al., 2004; Meredith and Westberry, 2004; Choi et al., 2005; Martinez et al., 2011; Keshavarzi et al., 2014). When taken together, our data that the NPSR-ir neurons in MePD and MePV receive the inputs from the OB and are also activated by NPS suggest that NPS system attenuates the defensive and risk assessment behaviors evoked by offensive odorants, at least in part, via action on the neurons of MePD and MePV of MeA.

NPS acts through a G protein coupled with the NPSR receptor (Xu et al., 2004). NPSR appears to be involved in the regulation of stress and anxiety (Reinscheid, 2008). Therefore, we wonder whether the activated neurons in the MePD and MePV induced by NPS bear NPSR, and the attenuating effects of NPS on the AP-evoked defensive and risk assessment behaviors are blocked by antagonist of NPSR. We found that i.c.v. administration of NPS enhanced Fos-ir neurons in the MePD and MePV, these neurons also expressed NPSR (Figure 6), and that [D-Val^5^]NPS, a selective antagonist of NPSR, blocked the effects of NPS on the AP-evoked defensive and risk assessment behaviors (Figure 4 and Supplementary Figure 1). In addition, the behavior characteristic of NPS and [D-Val^5^]NPS can be better replicated in MePD/MePV local injection experiment within lower dose (Figure 7 and Supplementary Figure 2). Collectively, these results suggest that NPS attenuates the anxiety-related behaviors evoked by AP through activation of the neurons bearing NPSR in the MePD and MePV of MeA, and strongly support that NPS-NPSR system plays an important role in anxiolysis.

In summary, our findings demonstrate that central action of NPS attenuates the AP-evoked defensive and risk assessment behaviors in mice. The neurons in the MePD and MePV of posterior medial amygdala receive the olfactory inputs containing AP signal from the OB, and bear NPSR and are activated by NPS. The effects of NPS on the AP-induced anxiety-related behaviors are blocked by selective antagonist of NPSR. Taken together, these findings indicate that NPS attenuates the AP-evoked defensive and risk assessment behaviors through activating the NPSR-expressing neurons in the posterior medial amygdala.

## Data Availability Statement

The original contributions presented in the study are included in the article/Supplementary Material, further inquiries can be directed to the corresponding author/s.

## Ethics Statement

The animal study was reviewed and approved by the Ethics Committee of Lanzhou University.

## Author Contributions

Y-FS, F-QX, and Y-PH contributed to the experimental design. Y-FS, CW, X-PR, H-DW, Y-LR, JL, C-YD, X-WY, and J-FX performed the experiments and analyzed the data. Y-FS and Y-PH interpreted the results and wrote the manuscript. All authors contributed to the article and approved the submitted version.

## Conflict of Interest

The authors declare that the research was conducted in the absence of any commercial or financial relationships that could be construed as a potential conflict of interest.

## Publisher’s Note

All claims expressed in this article are solely those of the authors and do not necessarily represent those of their affiliated organizations, or those of the publisher, the editors and the reviewers. Any product that may be evaluated in this article, or claim that may be made by its manufacturer, is not guaranteed or endorsed by the publisher.
