# Supplementary material for: Neuropeptide S Attenuates the Alarm Pheromone-Evoked Defensive and Risk Assessment Behaviors Through Activation of Cognate Receptor-Expressing Neurons in the Posterior Medial Amygdala
Source: Front Mol Neurosci. 2021 Dec 24;14:752516. doi: 10.3389/fnmol.2021.752516 (PMC8739225; doi:10.3389/fnmol.2021.752516)
Supplement: Supplementary file 1 [file Image_1.pdf]

Supplementary Material

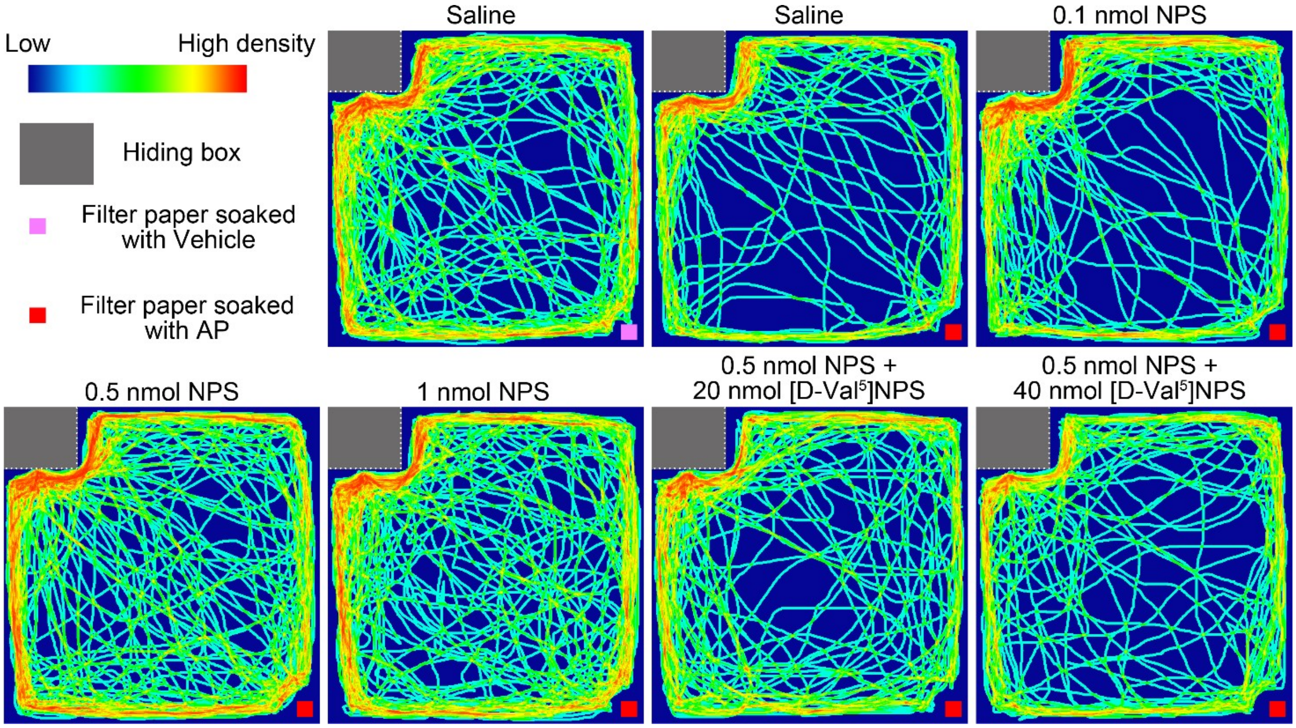

Supplementary Figure 1. Heatmap graphs of trajectory plots of all tested mice (n = 8 in each group) in figures 2–4. Color scaling from navy blue to red indicates the degree of overlap between trajectories in a particular cage region.

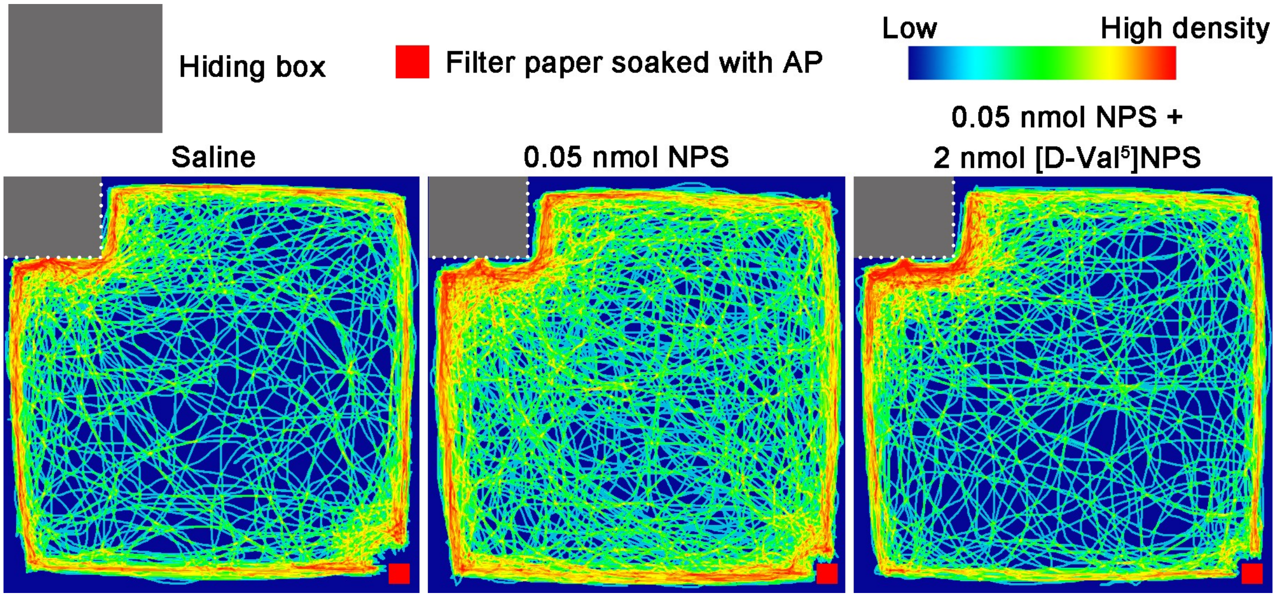

Supplementary Figure 2. Heatmap graphs of trajectory plots of all tested mice (n = 8 in each group) in figure 7. Color scaling from navy blue to red indicates the degree of overlap between trajectories in a particular cage region.
